# ‘Corona is coming’: COVID‐19 vaccination perspectives and experiences amongst Culturally and Linguistically Diverse West Australians

**DOI:** 10.1111/hex.13613

**Published:** 2022-10-19

**Authors:** Samantha J. Carlson, Gracie Edwards, Christopher C. Blyth, Barbara Nattabi, Katie Attwell

**Affiliations:** ^1^ Wesfarmers Centre of Vaccines and Infectious Diseases, Telethon Kids Institute Perth Western Australia Australia; ^2^ School of Social Sciences University of Western Australia Perth Western Australia Australia; ^3^ School of Population and Global Health University of Western Australia Perth Western Australia Australia; ^4^ School of Medicine University of Western Australia Perth Western Australia Australia

**Keywords:** attitudes, COVID‐19, ethnic and racial minorities, health knowledge, immunization, misinformation, vaccination

## Abstract

**Background:**

Culturally and Linguistically Diverse (CALD) groups within high‐income countries are at risk of being left behind by the COVID‐19 vaccination rollout. They face both access and attitudinal barriers, including low trust in government and health authorities.

**Objective:**

To explore perceptions and attitudes towards COVID‐19 vaccination, as well as facilitators, barriers and strategies to promote uptake among CALD residents of Western Australia (WA), where there were almost no COVID‐19 cases for 2 years.

**Design and Participants:**

Perth, WA's capital, was chosen as the state's study site because most of the state's CALD population lives there. Eleven semistructured in‐depth interviews and three focus groups (with 37 participants) were conducted with CALD residents between August and October 2021. Thematic analysis was conducted, informed by the ‘Capability’, ‘Opportunity’, ‘Motivation’, ‘Behaviour’ model.

**Results:**

CALD participants faced barriers including a lack of knowledge about COVID‐19 and the vaccines, low self‐rated English proficiency and education levels, misinformation, passive government communication strategies and limited access to vaccine clinics/providers. They were, however, motivated to vaccinate by the imminent opening of state and international borders, trust in government and healthcare authorities, travel intentions and the desire to protect themselves and others.

**Conclusions:**

Despite high levels of trust and significant desire for vaccines among CALD communities in Perth, current strategies were not meeting their needs and the community remains at risk from COVID‐19. Tailored intervention strategies are required to provide knowledge, address misinformation and facilitate access to ensure uptake of COVID‐19 vaccines—including for additional doses—amongst CALD communities. Governments should work with trusted CALD community members to disseminate tailored COVID‐19 vaccine information and adequately translated resources.

**Patient or Public Contribution:**

The Wesfarmers Centre of Vaccines and Infectious Diseases Community Reference Group at Telethon Kids Institute consulted on this project in September 2020; Ishar Multicultural Women's Health Services consulted on and facilitated the focus groups.

## 1 INTRODUCTION

Scholars and commentators recognize that even within high‐income countries, pockets of populations are at risk of being left behind in the globally inequitable COVID‐19 vaccine rollout.^1^ Culturally and Linguistically Diverse (CALD) populations, which include refugees and migrants, are at particular risk due to inaccessible or suboptimal healthcare experiences, which may compound attitudinal factors, including vaccine hesitancy.^2^, ^3^ Unique local factors can also create barriers: in the case of Western Australia (WA), almost nonexistent levels of community transmission of COVID‐19  led some locals to ‘wait awhile’ before being vaccinated as state borders remained closed throughout 2021.^4^ Meanwhile, earlier large outbreaks in other Australian states disproportionately affected CALD communities, with significantly higher morbidity and mortality.^5^, ^6^

In Australia, COVID‐19 vaccination is recorded on the Australian Immunisation Register, however, country of birth and language spoken at home are not captured on this register. Therefore, there is no publicly available data on uptake among CALD communities in Australia. However, an online survey of refugees in Australia in mid‐2021 identified that 88% had not yet received a COVID‐19 vaccine.^7^ Emerging evidence suggests that COVID‐19 vaccine hesitancy is prevalent among Australian CALD populations.^8^, ^9^ Focus groups conducted in mid‐2021 in New South Wales with CALD people indicated that 42% of participants were not planning to be vaccinated, and 29% were unsure or hesitant.^8^ Many had safety concerns, were uncertain how vaccines work or lacked trust in government.^8^ These uncertainties are unsurprising, given little of the public health information on COVID‐19 in Australia was tailored towards those with low English proficiency or health literacy.^10^ Lockyer et al.'s^11^ qualitative study in multicultural Bradford, UK, also found that participants' trust in government and the National Health Service dropped during the pandemic and that vaccine hesitancy was expressed by ethnic or national minorities.

In this study, we sought to understand the perceptions and attitudes to COVID‐19 vaccines amongst WA's CALD communities, to identify barriers preventing uptake, and to consider strategies to promote uptake in this population. Findings can support the development of strategies to enhance uptake among the CALD communities locally, nationwide and in comparable high‐income English‐speaking countries. They are particularly pertinent in the context of needing to enhance third and fourth‐dose rates for adults and protecting CALD children with paediatric COVID‐19 vaccinations.

## 2 METHODS

### 2.1 Study design

This CALD study was conducted as part of the larger mixed methods research project, Coronavax. Coronavax was initiated in April 2020 with the aim of ascertaining community attitudes towards vaccination and vaccine access in WA through a series of qualitative interviews with segmented population groups. A key aspect of the project involved translating findings to the state and federal governments in real‐time via Functional Dialogues, a novel formal research exchange mechanism in which data on community attitudes are presented to government officials, who then contribute their own reflections and experiences as additional research data (not reported here).^12^ Coronavax received ethics approval from the Child and Adolescent Services (CAHS) Human Research Ethics Committee (RGS0000004457).

### 2.2 Consumer consultation process

Consumer reference for Coronavax is led by Ms. Catherine Hughes who also chairs the Wesfarmers Centre of Vaccines and Infectious Diseases Community Reference Group at Telethon Kids Institute. This Group consulted on Coronavax in September 2020. Consumer involvement in Coronavax has sought to ensure the appropriateness and sensitivity of research questions and recruitment and to ensure that the research priorities and design reflect the needs of the community. Ishar Multicultural Women's Health Services, one of the largest multicultural health providers in Western Australia, provided additional consultation for this CALD study, as well as aiding with the recruitment of their clients, providing facilities, and hosting the focus groups.

### 2.3 Study setting

In the first 2 years of the COVID‐19 pandemic, WA experienced just over 1100 cases in a population of approximately 2.6 million people,^13^ with transmission rapidly increasing only from February 2022.^13^ As of March 2022, over 95% of people aged 12 years and older in WA had received at least two COVID‐19 vaccine doses,^14^ and over 85.5% of those eligible for their third dose in WA having received it.^15^ However, coverage rates at the time of our study were lower, as Australia had faced significant supply problems early in the rollout.^16^ Responsibility for vaccine operations sat with both State and Federal Governments. WA residents access COVID‐19 vaccines through general practice clinics, federal respiratory clinics, pharmacies, community health services and state‐led mass vaccination clinics.

As governments sought to rapidly increase vaccine uptake across the entire population, populations requiring more complex and bespoke interventions, such as CALD communities, remained under‐served. For example, Local Government Areas (LGAs) in Perth with the highest proportion of those born in nonmain English‐speaking countries had some of the lowest uptake in Perth 8 months into the rollout.^17^ A more tailored approach to CALD communities commenced in late 2021 after our study, including pop‐up vaccination clinics at major religious events (e.g., the Indian festival of Diwali, November 2021), or at multicultural community centres. The state‐wide campaign ‘Roll Up For WA’ translated resources into over 50 non‐English languages,^18^ although at the time of our study only approximately 20 were in place.

CALD individuals comprise almost one‐fifth of the WA population.^19^ In WA, 90% of individuals born in what the Australian Bureau of Statistics calls ‘non‐main English‐speaking countries’ reside in the Perth metropolitan region,^19^ so we focused on recruiting CALD adults living in Perth.

### 2.4 Sampling, recruitment and data collection

Interviewees were recruited through CALD organizations, social media (including a specific campaign directed towards CALD men), and promotional campaigns (in English) that ran from August to October 2021. Interviewees voluntarily signed up via the secure web‐based survey platform REDCap hosted by Telethon Kids Institute.^20^, ^21^ Here, they provided demographic and contact information. They were selected if they met the CALD definition and then contacted up to three times each via phone or email to organize the interview. The CALD definition was based on the Australian Bureau of Statistics' definition,^22^ and included individuals born overseas, having one or both parents born overseas from non‐English speaking countries, or whose first language is a Language Other Than English (LOTE). Interviews were conducted in English by SC over the phone or via teleconferencing software—interviewees who signed up all had sufficient English proficiency as the self‐sign‐up process was only in English.

Due to the high levels of English proficiency required for interview sign‐ups, we approached Ishar to partner on this research so that we could engage with CALD individuals who had lower English proficiency. Accordingly, focus group participants were recruited separately through their CALD community centre for women based in Stirling, the LGA with the highest proportion of individuals born in nonmain English‐speaking countries.^19^ Women attending regular English classes were invited face‐to‐face by researchers and their teachers to participate in a focus group in lieu of their class. Focus groups were run face‐to‐face by S. J. C., G. E. and K. A. in the CALD community centre; they were conducted in English with the use of peer translators for participants with low English proficiency.

Data were collected between August and October 2021. Of the 23 CALD individuals who voluntarily signed up online to participate in the individual interviews, we interviewed 11, with the remaining 12 being lost to follow‐up. We ran three focus groups at the Ishar multicultural centre with their female clients, comprised of 6–16 participants per group (total *N* = 37). All women who were present consented and participated in a focus group—there were no refusals. Interviews and focus groups lasted approximately 60 min. Of the total 48 participants, 90% were female and 29% were aged 30–39 years. Approximately, one third (35%) had been to university, 54% self‐reported high English proficiency and 27% reported having received at least their first COVID‐19 vaccine (Table 1). Further, 46% had migrated to Australia since 2010, 35% were born in Southern Asia, and 75% identified with a religion (most common being Islam and Christianity). Two of the 11 interviewees self‐identified as community leaders. Interviewees gave voluntary, informed, written consent; focus group participants gave voluntary, informed, verbal consent. All have been assigned pseudonyms.

**Table 1:** Sociodemographic characteristics of the 48 CALD participants, Perth, WA

Demographics	Interviewees number (%)	Focus group participants number (%)	All participants number (%)
COVID‐19 vaccine status (self‐reported)			
Vaccinated	10 (91)	3 (8)	13 (27)
Unvaccinated	1 (9)	26 (70)	27 (56)
Unknown	0 (0)	8 (22)	8 (17)
Age (years)			
18–29	3 (27)	5 (14)	8 (17)
30–39	2 (18)	12 (32)	14 (29)
40–49	1 (9)	6 (16)	7 (15)
50–59	3 (27)	4 (11)	7 (15)
60+	2 (18)	3 (8)	5 (10)
Unknown	0 (0)	7 (19)	7 (15)
Gender			
Female	6 (55)	37 (100)	43 (90)
Male	5 (45)	0 (0)	5 (10)
Subregion of origin			
Eastern Asia	1 (9)	2 (5)	3 (6)
Middle Africa	0 (0)	1 (3)	1 (2)
Northern Africa	0 (0)	1 (3)	1 (2)
South‐eastern Asia	8 (73)	1 (3)	9 (19)
Southern Asia	1 (9)	16 (43)	17 (35)
Southern Europe	1 (9)	1 (3)	2 (4)
Sub‐Saharan Africa	0 (0)	6 (16)	6 (13)
Western Asia	0 (0)	2 (5)	2 (4)
Unknown	0 (0)	7 (19)	7 (15)
Year of arrival to Australia			
Before 1990	1 (9)	0 (0)	1 (2)
1990–1999	3 (27)	0 (0)	3 (6)
2000–2009	2 (18)	9 (24)	11 (23)
2010–2019	4 (36)	18 (49)	22 (46)
Unknown	1 (9)	10 (27)	11 (23)
Language spoken at home^a^			
Amharic	0 (0)	2 (5)	2 (4)
Arabic	0 (0)	7 (19)	7 (15)
Cantonese	1 (9)	1 (3)	2 (4)
English	7 (64)	4 (11)	11 (23)
Farsi	1 (9)	10 (27)	11 (23)
Swahili	0 (0)	2 (5)	2 (4)
Tagalog	2 (18)	0 (0)	2 (4)
Unknown/other^b^	5 (45)	17 (46)	22 (46)
Religion			
Bahai	0 (0)	1 (3)	1 (2)
Buddhism	0 (0)	1 (3)	1 (2)
Christian	5 (45)	7 (19)	12 (25)
Hinduism	0 (0)	1 (3)	1 (2)
Islam	2 (18)	19 (51)	21 (44)
No religion	4 (36)	2 (5)	6 (13)
Unknown	0 (0)	6 (16)	6 (13)
Self‐rated English proficiency			
Very well	11 (100)	5 (14)	16 (33)
Well	0 (0)	10 (27)	10 (21)
Not well/not at all	0 (0)	14 (38)	14 (29)
Not at all	0 (0)	1 (3)	1 (2)
Unknown	0 (0)	8 (22)	8 (17)
Highest level of education			
Primary school	0 (0)	4 (11)	4 (8)
Secondary school	0 (0)	9 (24)	9 (19)
TAFE/apprenticeship	0 (0)	9 (24)	9 (19)
University degree	11 (100)	6 (16)	17 (35)
No formal education	0 (0)	4 (11)	4 (8)
Unknown	0 (0)	5 (14)	5 (10)

Abbreviations: CALD, Culturally and Linguistically Diverse; TAFE, Technical and Further Education; WA, Western Australia.

^a^ Some participants spoke ≥1 language.

^b^ Other languages include Acholi, Azeri, Bomwali, Hazaragi, Hindi, Ilocano, Indonesian, Italian, Kikuyu (Bantu), Korean, Macedonian, Malay, Oromo, Somali, Tedim, Urdu and Vietnamese.

### 2.5 Interview guide

Interview guides were developed by the multidisciplinary (project) team comprised of vaccination, medical, policy, social science and government scholars, and an indicative guide is published open‐access with our protocol.^12^ The questions were simplified for the focus groups.

### 2.6 Analysis

All interviews and focus groups were audio‐recorded and professionally transcribed verbatim. Thematic analysis followed the Braun and Clarke method,^23^ with deductive analysis in NVivo 12 using the Capability, Opportunity, Motivation, Behaviour (COM‐B) model.^24^ We selected COM‐B as it is used extensively by the World Health Organization to understand reasons for (non)vaccination among specific populations.^25^ G. E. generated initial codes, S. J. C. and B. N. each coded a manuscript to provide additional perspectives, including a CALD lived experience lens, and the three coders revised the matrix via a collaborative meeting before GC applied it to the full data set.

## 3 FINDINGS

**Figure 1:**
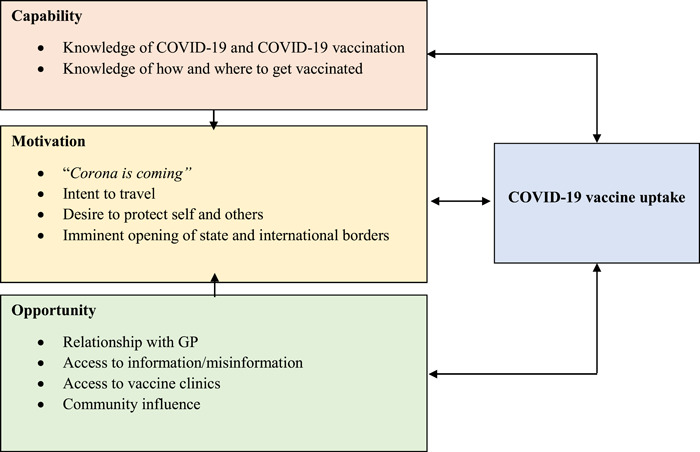
The Capability, Opportunity and Motivation influencing COVID‐19 vaccine uptake among Culturally and Linguistically Diverse individuals in Perth, WA. Figure modified from COM‐B model^24^; arrows indicate influence of factor on other factors.

Drawing on data from our interviews and focus groups, we created 10 subthemes encompassed within the three superordinate themes of Capability, Opportunity and Motivation (Figure 1).

### 3.1 Capability

#### 3.1.1 Knowledge about COVID‐19 and COVID‐19 vaccination

Most participants lacked an understanding of the potential severity of COVID‐19 infection, transmission routes, and vaccine ingredients or efficacy. This was particularly expressed by female focus group participants who had low English proficiency and education levels. Zi (Female, age unknown) said:

I want to know, if you have your vaccines, how you know that you're protected from the virus?

Some were also unsure whether the COVID‐19 vaccine brands being used in Australia would be accepted by their home country. Further, many with pre‐existing health conditions feared severe side effects from the vaccines:

She has her two [adult] boys back in Sudan. She applied for them to come here 7–8 years ago, but they [the Australian government] didn't accept them in … and now she has so many health problem … she's scared if she take [the vaccine] … maybe something will happen to her [like death] and she will not see her [adult] kids … she doesn't know what is right—(Sita; focus group attendee providing real‐time peer translation for Nell [Female, 70 years old])

#### 3.1.2 Knowledge about how and where to get vaccinated

Though many participants knew they could receive a vaccine at a mass vaccination clinic, few were aware they could be vaccinated at a general practice clinic. Many also did not know vaccination was free. Finally, some did not know how to navigate the online booking system; a few had tried, but with no success:

Yes, I try yesterday to do online … doesn't work with me—(Ezma [Female, 40 years]; Focus Group)

### 3.2 Opportunity

#### 3.2.1 Relationships with general practitioners (GPs)

Many participants had GPs who spoke their language and/or were CALD, leading to strong trust in their recommendations:

If my doctor said [COVID‐19 vaccination] was good, I would get it—(Hana [Female, age unknown]; Focus Group)

Although many participants were unvaccinated, the appeal of convenient, trustworthy, and familiar healthcare providers meant they would choose to be vaccinated at their general practice clinics rather than travelling to mass clinics. Participants also felt more comfortable asking their CALD GP questions about the vaccine, compared to unfamiliar providers who don't speak their language. They also trusted that their GPs “don't want to kill us”. However, not all participants had established comfortable relationships with GPs, and some were concerned that GPs lack the cultural awareness skills or resources to communicate with participants.

…some of our [CALD] community members have got their own GPs that they trust. Some [general practice clinics are] just like going to a factory—(Reyna [Female, 53 years]; Community Leader, Interview)

#### 3.2.2 Access to information/misinformation

Participants cited significant concerns about COVID‐19 vaccine safety, often informed by misinformation. They depicted their immersion within tight‐knit communities, sharing information at gatherings, informal community events and social interactions. Friends and contacts in countries of origin or in other destination countries also spread (mis)information, including through social media.

…she doesn't have any ideas about the vaccine. And she hearing so many, you know, gossip around … she doesn't know what is the right—(Sita; focus group attendee providing real‐time peer translation for Nell [Female, 70 years old])

‘Gossip’ was centred on safety: people were discussing contracting COVID‐19 from the vaccine, or extreme vaccine side effects including infertility, memory, cognition issues and death occurring specifically 2 years after vaccine receipt:

…almost everyone, they talking about, like, you know, the injection for the COVID, if they take it or not. And everyone said, ‘Don't take it! You're going to die! You're going to die!’—(Ezma [Female, 40 years]; Focus Group)

CALD individuals wanted to protect themselves against COVID‐19, but not always through vaccination, given these safety fears. Alternatives to vaccination—seen as being risk‐free—were actively shared among community members:

My mum's side [of the family] started sending … WhatsApp messages … ‘Oh, if you take an onion and you cut it in half … leave it in a corner of your house, all the coronavirus is going to go to the onion and you'll be safe’—(Inda [Female, 23 years]; Interview)

Some participants relied on WA government online resources and mainstream media (predominantly broadcast in English only) such as Channels Seven, Nine and the Australian Broadcasting Corporation (ABC). Few were aware of or consumed media in their language distributed by the Special Broadcasting Service (SBS), the main multilingual broadcaster in Australia. Most participants with no/low English proficiency relied heavily on social media (mostly Facebook or WhatsApp) and sourced information in their preferred language, shared by internationally residing relatives or their local CALD community.

Even though my mum can read Chinese … she wouldn't be actively going to websites to check, and she would be relying on her daughters or friends to tell her what's happening—(Liling [Female, 50 years]; Interview)

Some participants spoke multiple languages but still expressed a preference to consume vaccination information in only one of them—that being their first language.

Some participants sought accurate information from their doctors; however, their vaccine‐related fears were not always allayed.

…she went to the doctor but … the doctor just want to give her the vaccine. But for herself, she's not sure, you know, she confused. She want the doctor to check everything before she takes the vaccine—(Sita [Female, 29 years]; focus group attendee providing real‐time peer translation for Nell [Female, 70 years old])

Several multicultural organizations and community leaders seeking to provide accurate information attempted to collaborate with government agencies or coordinate seminars engaging the community in their own environments.

…she [CALD public health academic] came to the centre and gave talks about the vaccination … that's a good way of doing it—(Susi [Female, 50 years]; Interview)

However, some community leaders were themselves sharing COVID‐19 vaccine misinformation on social media:

On Facebook … even the priests and bishops are actually announcing to their parishioners the [the] vaccine is not good. (Reyna [Female, 53 years]; Community Leader, Interview)

Participants were disappointed in government attempts to access and work with their communities. There were no facilitation programmes in CALD communities, who lacked resources and noted little lingual or visual diversity in promotional campaigns.

[Create] ‘Roll Up Your Sleeve’ [resources] in Arabic or ‘Roll Up Your Sleeve’ [resources] in Chinese. Not just English—(Kasih [Female, 50 years]; Interview)

Why not approach [CALD communities] instead of asking them to come in? Because it means we give them … practical plan … the [CALD] community people … [the WA government] cannot wait for the ball, [they] need to also try to get the ball—(Audrey [Female, 43 years]; Interview)

Participants believed that governments needed to be more active in reaching out to them, and said they would welcome efforts made by government and health representatives to come to the community to talk about COVID‐19 vaccination.

#### 3.2.3 Access to vaccine clinics

Locations of mass COVID‐19 vaccination clinics were inconvenient for participants, given a lack of transport options, particularly as some could not drive. In an interview, Susi said:

Even [the mass vaccination centre] in the city [Perth convention centre] … [it's] too hard, how will people go!?

Other participants feared leaving their perceived safe and COVID‐19‐free community to travel into an unfamiliar part of Perth.

My wife she was really, really panicked [to leave the house] … she was kind of anxious. Same for my mum—(Tai [Male, 29 years]; Interview)

For participants who did get vaccinated, the most convenient locations were clinics at or close to their workplaces or educational institutions. Some did attend mass vaccination clinics and found walk‐in processes highly convenient and well‐facilitated.

#### 3.2.4 Community influence

Participants had high levels of trust in their own communities; opinions and experiences shared by peers were greatly valued and reassuring.

In the community, we were very, very scared. But after that, when I saw friend and family [had been vaccinated], a couple of people they do fine, they are perfectly fine. My friend, she's a doctor. She done vaccine last month … her husband and other colleague did it, but they got a fever … they took Panadol after … is fine—(Momo [Female, 37 years]; Focus Group)

However, the sharing of misinformation and negative opinions created situations in which some participants were torn between what their community was saying and the professional medical advice:

When I saw, yesterday, the doctor, everybody comes to me … say don't do this … But in my heart, I say, ‘No, I'm going to take it!’—(Ezma [Female, 40 years]; Focus Group)

Some participants felt shame around being vaccinated, and sought to avoid undue attention or stigma from hesitant friends/family who were concerned that vaccinated people would die in 2 years:

People … said ‘be nice with them, they going to live only two years … After two years they will die’. (Momo [Female, 37 years]; Focus Group)

Of the two community leaders we interviewed, one was completely against the idea of any medical intervention, including vaccination:

I've never taken any flu vaccines, I never take any painkillers, I avoid doctors like the plague … I want my body to learn how to cope and how to fight those diseases … And if I can't cope, I will simply go to the next world—(Ubel [Male, 78 years]; Interview)

Ubel volunteers at a community organization for refugees and migrants in Perth. His role is to share information with those in the database. When asked whether he shares information on COVID‐19 vaccination, he said:

We get a quite a number [of emails from various official and unofficial sources] and I've passed on. Except yesterday for the first time, I did not send one on because this [particular] group sent me an email and they are totally convinced about COVID and it was a bit too strong for my liking. So I did not send it, but that's the first one.

Some participants also discussed the role of community leaders, saying these leaders did not promote that they were vaccinated, fearing negative feedback or loss of power:

[If community leaders] had a vaccine, they don't tell people, their own group … because other people … look up to them and … most of us don't believe in [the vaccine]—(Reyna [Female, 53 years]; Community Leader, Interview)

Reyna was a pro‐vaccination community leader, and she expressed frustration in feeling solely responsible for vaccine uptake in her wider CALD community. She felt the government had not come to her organization to provide support; community leaders instead had to organize seminars themselves with health representatives to answer community questions.

We're not the only not‐for‐profit community centre that are not well informed of the COVID and now [the WA government is] asking us to ‘roll up’ … how could you have a vaccine if you weren't knowledgeable, you have no education about it at all?—(Reyna [Female, 53 years]; Community Leader, Interview)

### 3.3 Motivation

Most participants were “scared”, “confused” or “unsure” about COVID‐19 vaccines, particularly because, as Umme said in a focus group “this medicine only come in 2 years, it come quickly, why so quickly, maybe something wrong”. Yet despite these concerns, many were nevertheless willing to be vaccinated to protect themselves, their family and their community.

I'm getting this so that others around me don't get sick. I'm getting this so that I can protect my mum, I can protect my dad, I can protect my friends—(Angelo [Male, 37 years]; Interview)

Participants, aware that “Corona is coming”, felt they would be at greater risk once international and interstate borders opened, declaring that they would get vaccinated once WA borders opened (but likely not before). Others said the border opening would mean they could visit their friends and family overseas; they believed COVID‐19 vaccination would be a requirement for this:

I want to go visit my family [in Ethiopia] … But [also] I'm happy that we are closed here. Because then we … stay safe—(Mahsa [Female, 34 years]; Focus Group)

## 4 DISCUSSION

This is the first study to explore perceptions and attitudes to the COVID‐19 vaccine, barriers to preventing and strategies promoting vaccine uptake among CALD populations in Perth, WA. Similar to studies from the United Kingdom^11^, ^26^ and New South Wales,^8^ we found high levels of COVID‐19 vaccine hesitancy among CALD individuals due to safety concerns, lack of understanding and high levels of misinformation perpetuated by CALD peers or social media platforms. Hesitancy and low uptake were particularly apparent among participants with low levels of education and low English proficiency. These findings have since been confirmed in a large regression analysis of over 12 million Australians, which identified that those with low levels of education and English proficiency were less likely to have received their first COVID‐19 vaccine dose.^27^

Despite their concerns, participants were not completely opposed to vaccination, and we were struck by how they did not see the COVID‐19 vaccination programme as being for people like them. They were exposed to misinformation through their social networks, as previous studies have also found,^11^ and the hesitancy this generated is a wider problem. However, participants also felt that governments were not forthcoming with resources to address their concerns. Participants were ostensibly included in cohorts that were eligible for vaccination at the time of data collection, and some of them had been eligible many months prior, due to their age. However, although they were ostensibly being asked to ‘roll up’, they received few or no personal cues to avail themselves of vaccinations.

Practical barriers were also evident. As previously identified by Abdi et al.,^28^ refugees and migrants tend to have quite demanding competing priorities, such as seeking employment and accommodation and thus need prompting for vaccination. In some cases, our participants' efforts to be vaccinated were thwarted by the online booking system. In fact, some of our research team assisted participants to book vaccination appointments on their smartphones after the focus groups or provide further information to partners who waited in the car park to speak with them. It is not just our participants who struggled with the online booking system: Seale et al.^29^ describe a ‘digital divide’ in which those without access to the internet and have low English proficiency are disadvantaged in their ability to access COVID‐19 vaccines, and Biddle et al.^27^ identified that Australians who completed the 2016 census on paper rather than online had a lower probability of vaccination, suggesting that individuals with lower digital literacy may experience more barriers to vaccination. Booking systems must be accessible to varying levels of digital literacy and English proficiency.

Social norms are a strong influence on vaccine uptake.^16^, ^30^ For CALD people in Perth, social norms were blocking COVID‐19 vaccination. Vaccinated CALD individuals were not sharing their experiences or reasons for vaccinating, facilitating ongoing shame and stigma, and thereby perpetuating continued silence for those who did vaccinate; tendencies that have also been found in local ‘alternative’ communities with regard to childhood vaccination.^31^ Some CALD leaders were also not actively encouraging vaccination due to their own concerns or opposition, an issue identified early on by the Australian multicultural sector as a potential barrier.^32^

Another well‐established influence on vaccine uptake is receiving a recommendation from a healthcare provider.^30^ Our participants showed great trust in their GPs and reported positive experiences of consultation. Accordingly, they believed that GPs would have more opportunities to address COVID‐19 vaccine concerns than mass vaccination clinic staff. However, GPs may not always be easily accessible within an acceptable timeframe to the patient,^33^ meaning some CALD individuals are turning to or relying on social media for their health/COVID‐19 information.^9^

Our participants' high levels of trust in governing authorities was a novel finding, mostly related to feeling safe from COVID‐19 in WA. Focus groups with CALD individuals in New South Wales instead found participants were concerned that the government was using COVID‐19 vaccination to control people.^8^ We were struck by how our participants' trust in government emanated from people whose experiences in their countries of origin had often been traumatic, culminating in their refugee status.

## 5 IMPLICATIONS FOR POLICY AND PRACTICE

A strong motivation for this research was to assist in strategic design, informing ongoing and future implementation of public health initiatives among CALD populations. Our experiences engaging with our CALD participants and their partners, including helping them to book appointments after the focus groups, emphasized how important it is that governments promptly address vaccine access issues, as well as campaigning and working directly with CALD groups, and featuring them visibly in messaging. The barriers regarding lack of access to resources and the circulation of misinformation must be addressed as soon as possible to reduce the COVID‐19 burden amongst the CALD population in WA. These lessons can then be utilized for future vaccine doses, for reaching the children of CALD populations, and for wider public health initiatives.

At the time of our study, CALD participants and their communities were not publicly sharing their vaccinated status and proudly promoting COVID‐19 vaccinations. However, it is unreasonable to demand more social courage from CALD individuals when what they need is more support. Given that governments are responsible for making vaccination accessible and acceptable to improve and sustain uptake,^34^ they must recognize, work with, and train influential community leaders on the importance of and how to have meaningful vaccine conversations with their peers.^32^ A recent review identified the who, the what and the how for effective community engagement during a pandemic: essentially, it is important to engage with the community from the very beginning for a better chance of behaviour change within the community.^35^ In eastern Australia, Mahimbo et al.^9^ identified that CALD communities are eager for community engagement, and for their leaders to champion the vaccine. Seale et al.^29^ identified that while some CALD organizations in Australia were updating local CALD leaders on relevant COVID‐19 information to share with their community, there was a lack of training in how to do so. Training influential community leaders can occur over a relatively short period of time: a COVID‐19 information and support programme was offered in the United States to CALD community leaders which involved 1‐hour conference calls twice a week (we acknowledge that this is then reliant on the leaders having a decent level of computer literacy.)^29^ Further, given the trust and respect our participants held for their health practitioners, it is imperative that governments support GPs to effectively communicate about COVID‐19 vaccination with CALD patients; this requires resources, training and remuneration.^36^ Since our data collection, the Australian National Centre for Immunisation Research and Surveillance have published a COVID‐19 decision aid in Greek, Vietnamese, Arabic, Simplified Chinese and Traditional Chinese.^37^ Immunisation providers could use or share this tool with their CALD patients (though its effectiveness in increasing vaccine uptake is yet to be evaluated).

Our participants' high levels of trust in governing authorities is an untapped opportunity. WA participants knew that ‘Corona is coming’. Now that it has arrived, the government and healthcare providers must maintain the trust of CALD by reaching them promptly with vaccine doses and culturally appropriate information to reduce the burden of COVID‐19. If these measures fail, it would be entirely reasonable for CALD individuals to not blame their own choices, and instead look to the authorities they believed would protect them and ask why they were not offered greater support and access.

### 5.1 Limitations

Qualitative data is not widely generalizable, as its function is to generate deep insights when little is known about an issue, or when data cannot be captured appropriately via quantitative means, including across language barriers. Different Australian states had different pandemic experiences and our data may not be applicable to other states or countries, although our insights and reflections remain pertinent. Our interviews and focus groups took place well into the COVID‐19 pandemic and vaccine rollout, but before community transmission in WA—the subsequent ‘first wave’ from February 2022 may have changed participants' views. Our recruitment method for interviews skewed demographics towards those with high English proficiency, females (despite a social media campaign specifically recruiting men), high levels of education and high COVID‐19 vaccine uptake. However, we mitigated this by collaborating with a community centre for recent migrants/refugees to undertake focus groups with women possessing low levels of English proficiency, education, and COVID‐19 vaccine uptake. Participants' language constraints were mitigated by strong peer relationships that facilitated peer translation. Despite our efforts to mitigate these limitations, our study does not represent the entire CALD community in Perth.

## 6 CONCLUSION

This study provides in‐depth insight into the perceptions and attitudes of CALD communities in Perth, WA, regarding the COVID‐19 vaccine, identifying facilitators and barriers for engagement and uptake. CALD participants faced barriers including a lack of knowledge about COVID‐19 and the vaccines, low English proficiency and education levels, misinformation, passive government communication strategies and limited access to vaccine clinics/providers. They were, however, motivated to vaccinate by the imminent opening of state and international borders, trust in government and healthcare authorities, travel intentions and the desire to protect themselves and others. Despite high levels of trust and significant desire for vaccines, strategies at the time of our study were not meeting the CALD community's needs, and thus those within it remain at risk from the disease. Tailored intervention strategies are required to provide knowledge, address misinformation, and facilitate access to ensure uptake of COVID‐19 vaccines—including for additional doses—amongst CALD communities. Governments must work with trusted CALD community members and immunization providers such as GPs to disseminate tailored COVID‐19 vaccine information and adequately translated resources so that community members themselves can become stronger vaccine ambassadors to each other.

## AUTHOR CONTRIBUTIONS

Samantha J. Carlson supervised the data collection and analysis, led the initial manuscript drafting, contributed to the study design, contributed to project administration and coordinated the ethics and stakeholder engagement. Gracie Edwards collected the data and conducted the analysis as well as drafted the literature review and contributed to the overall manuscript drafting. Christopher C. Blyth contributed to the conceptualization of the project and provided oversight and input into the study and funding attainment, and reviewed and edited the manuscript. Barbara Nattabi supervised the data collection and analysis, contributed to the coding framework, provided CALD expertise, contributed to project administration and reviewed and edited the manuscript. Katie Attwell conceptualized the project, co‐developed the broader study methodology with Christopher C. Blyth, led the funding attainment, and supervised the conduct and administration of the broader research project. She contributed to the literature review and the drafting, reviewing and editing of the manuscript. All authors have approved the final manuscript.

## CONFLICT OF INTEREST

K. A. and C. C. B. are specialist advisors to the Australian Technical Advisory Group on Immunisation. The remaining authors declare no conflict of interest.

## Data Availability

Research data are not shared.

## References

[1] Mukumbang FC . Are asylum seekers, refugees and foreign migrants considered in the COVID‐19 vaccine discourse. BMJ Glob Health. 2020;5(11):1‐4. 10.1136/bmjgh-2020-004085 PMC7661346 33177039

[2] Crawshaw AF , Farah Y , Deal A , et al. Defining the determinants of vaccine uptake and undervaccination in migrant populations in Europe to improve routine and COVID‐19 vaccine uptake: a systematic review. Lancet Infect Dis. 2022;22:1‐13. 10.1016/S1473-3099(22)00066-4 35429463 PMC9007555

[3] P Iqbal M, P , Walpola R , Harris‐Roxas B , et al. Improving primary health care quality for refugees and asylum seekers: a systematic review of interventional approaches. Health Expect. 2021:1‐30. 10.1111/hex.13365 PMC9615090 34651378

[4] Carlson SJ , McKenzie L , Roberts L , Blyth CC , Attwell K . Does a major change to a COVID‐19 vaccine program alter vaccine intention? A qualitative investigation. Vaccine. 2022;40(4):594‐600. 10.1016/j.vaccine.2021.12.021 34952758 PMC8674511

[5] Australian Bureau of Statistics . COVID‐19 mortality in Australia. 2022. Accessed April 1, 2022. https://www.abs.gov.au/articles/covid-19-mortality-australia#death-due-to-covid-19-country-of-birth

[6] Roder C , Maggs C , McNamara BJ , et al. Area‐level social and economic factors and the local incidence of SARS‐CoV‐2 infections in Victoria during 2020. Med J Aust. 2021;216:349‐356. 10.5694/mja2.51436 PMC9115064 35224751

[7] Liddell BJ , Murphy S , Mau V , et al. Factors associated with COVID‐19 vaccine hesitancy amongst refugees in Australia. Eur J Psychotraumatol. 2021;12(1):1‐4. 10.1080/20008198.2021.1997173 34868488 PMC8635584

[8] Social Equity Works . *Issues, Barriers and Perceptions About the COVID‐19 Vaccine Among Culturally and Linguistically Diverse Communities in NSW*. NSW Council of Social Service; 2021. Accessed March 7, 2022. https://www.ncoss.org.au/policy-advocacy/policy-research-publications/issues-barriers-and-perceptions-about-the-covid-19-vaccine-among-culturally-and-linguistically-diverse-communities-in-nsw/

[9] Mahimbo A , Kang M , Sestakova L , Smith M , Dawson A . Factors influencing refugees' willingness to accept COVID‐19 vaccines in Greater Sydney: a qualitative study. Aust N Z J Public Health. 2022;46(4):502‐510. 10.1111/1753-6405.13252 35555951 PMC9347689

[10] Mac OA , Muscat DM , Ayre J , Patel P , McCaffery KJ . The readability of official public health information on COVID‐19. Med J Aust. 2021;215(8):373‐375. 10.5694/mja2.51282 34580878 PMC8661844

[11] Lockyer B , Islam S , Rahman A , et al. Understanding COVID‐19 misinformation and vaccine hesitancy in context: findings from a qualitative study involving citizens in Bradford, UK. Health Expect. 2021;24(4):1158‐1167. 10.1111/hex.13240 33942948 PMC8239544

[12] Attwell K , Carlson S , Tchilingirian J , et al. Coronavax: preparing community and government for COVID‐19 vaccination: a research protocol for a mixed methods social research project. BMJ Open. 2021;11(6):e049356. 10.1136/bmjopen-2021-049356 PMC8249174 34193501

[13] Government of Western Australia: Department of Health . Coronavirus COVID‐19 in Western Australia. 2022. Accessed April 29, 2022. https://experience.arcgis.com/experience/359bca83a1264e3fb8d3b6f0a028d768

[14] WA Government Department of Health . COVID‐19 coronavirus: vaccination dashboard. 2022. Accessed April 1, 2022. https://www.wa.gov.au/government/covid-19-coronavirus/covid-19-coronavirus-vaccination-dashboard

[15] Australian Government: Operation COVID Shield . COVID‐19 vaccine rollout. 2022. Accessed April 1, 2022. https://www.health.gov.au/sites/default/files/documents/2022/03/covid-19-vaccine-rollout-update-8-march-2022_0.pdf

[16] Kaufman J , Tuckerman J , Danchin M . Overcoming COVID‐19 vaccine hesitancy: can Australia reach the last 20 percent. Expert Rev Vaccines. 2021;21:159‐161. 10.1080/14760584.2022.2013819 34854334

[17] Shine R . Perth's most affluent suburbs surging ahead with COVID‐19 vaccination rates. *ABC News*. 2022. Accessed April 1, 2022. https://www.abc.net.au/news/2021-10-12/perth-covid-19-vaccination-rate-suburb-breakdown/100530756

[18] Government of Western Australia . COVID‐19 coronavirus: translated information on vaccination. 2022. Accessed August 16, 2022. https://www.wa.gov.au/government/covid-19-coronavirus/covid-19-coronavirus-translated-information-vaccination

[19] Government of Western Australia: Department of Local Government, Sport and Cultural Industries: Office of Multicultural Interests . Western Australians from Culturally and Linguistically Diverse backgrounds: a profile. 2021. Accessed April 1, 2022. https://www.omi.wa.gov.au/docs/librariesprovider2/statistics/wa-cald-profile-2021.pdf?sfvrsn=a635fb38_6

[20] Harris PA , Taylor R , Thielke R , Payne J , Gonzalez N , Conde JG . Research electronic data capture (REDCap)—a metadata‐driven methodology and workflow process for providing translational research informatics support. J Biomed Inf. 2009;42(2):377‐381. 10.1016/j.jbi.2008.08.010 PMC2700030 18929686

[21] Harris PA , Taylor R , Minor BL , et al. The REDCap consortium: building an international community of software platform partners. J Biomed Inf. 2019;95(103208):1‐10. 10.1016/j.jbi.2019.103208 PMC7254481 31078660

[22] Australian Bureau of Statistics . Standards for statistics on Cultural and Language Diversity. 1999. Accessed April 1, 2022. https://www.abs.gov.au/ausstats/abs@.nsf/mf/1289.0

[23] Braun V , Clarke V . Using thematic analysis in psychology. Qual Res Psychol. 2006;3(2):77‐101. 10.1191/1478088706qp063oa

[24] Michie S , Van Stralen MM , West R . The behaviour change wheel: a new method for characterising and designing behaviour change interventions. Implement Sci. 2011;6(1):1‐12. 10.1186/1748-5908-6-42 21513547 PMC3096582

[25] World Health Organization . Tailoring Immunization Programmes (TIP). 2020. Accessed April 1, 2022. https://www.who.int/europe/activities/tailoring-immunization-programmes-(tip)

[26] Razai MS , Osama T , McKechnie DG , Majeed A . Covid‐19 vaccine hesitancy among ethnic minority groups. BMJ. 2021;372(n513):1‐2. 10.1136/bmj.n513 33637577

[27] Biddle N , Welsh J , Butterworth P , Edwards B , Korda R . Socioeconomic determinants of vaccine uptake: July 2021 to January 2022. ANU COVID‐19 Vaccine Series. 2022. Australian National University. Accessed August 8, 2022. https://www.health.gov.au/resources/publications/socioeconomic-determinants-of-vaccine-uptake-july-2021-to-january-2022

[28] Abdi I , Menzies R , Seale H . Barriers and facilitators of immunisation in refugees and migrants in Australia: an east‐African case study. Vaccine. 2019;37(44):6724‐6729. 10.1016/j.vaccine.2019.09.025 31537444

[29] Seale H , Harris‐Roxas B , Heywood A , et al. Speaking COVID‐19: supporting COVID‐19 communication and engagement efforts with people from culturally and linguistically diverse communities. BMC Public Health. 2022;22(1):1‐11. 10.1186/s12889-022-13680-1 35761264 PMC9235158

[30] Brewer NT , Chapman GB , Rothman AJ , Leask J , Kempe A . Increasing vaccination: putting psychological science into action. Psychol Sci Public Interest. 2017;18(3):149‐207. 10.1177/1529100618760521 29611455

[31] Attwell K , Meyer S , Ward P . The social basis of vaccine questioning and refusal: a qualitative study employing Bourdieu's concepts of ‘Capitals’ and ‘Habitus’. Int J Environ Res Public Health. 2018;15(5):1‐17. 10.3390/ijerph15051044 PMC5982083 29789482

[32] Seale H , Harris‐Roxas B , Heywood A , et al. The role of community leaders and other information intermediaries during the COVID‐19 pandemic: insights from the multicultural sector. Humanit Soc Commun. 2022;9(174):1‐7. 10.1057/s41599-022-01196-3

[33] Australian Institute of Health and Welfare . Patient experiences in Australia by small geographic areas in 2019–20. 2021. Accessed August 16, 2022. https://www.aihw.gov.au/reports/primary-health-care/patient-experiences-small-geographic-areas-2018-19/contents/patient-experiences-in-australia-by-phn

[34] Attwell K , Hannah A , Leask J . *COVID‐19: Talk of ‘vaccine hesitancy’ Lets Governments off the Hook*. Nature; 2022. Accessed April 1, 2022. https://www.nature.com/articles/d41586-022-00495-8 10.1038/d41586-022-00495-8 35194212

[35] Gilmore B , Ndejjo R , Tchetchia A , et al. Community engagement for COVID‐19 prevention and control: a rapid evidence synthesis. BMJ Glob Health. 2020;5(10):1‐11. 10.1136/bmjgh-2020-003188 PMC7554411 33051285

[36] Mahimbo A , Abdi I , Heywood A . *COVID‐19 Vaccine Uptake in CALD Communities: Support GPs Better*. MJA Insight; 2021. Accessed April 1, 2022. https://insightplus.mja.com.au/2021/34/covid-19-vaccine-uptake-in-cald-communities-support-gps-better/

[37] National Centre for Immunisation Research and Surveillance . Decision aid (16+ years): should I get the COVID‐19 vaccine? 2021. Accessed August 8, 2022. https://www.ncirs.org.au/covid-19-decision-aid-for-adults

